# HIF-3α1 promotes colorectal tumor cell growth by activation of JAK-STAT3 signaling

**DOI:** 10.18632/oncotarget.7272

**Published:** 2016-02-09

**Authors:** Xiang Xue, Kylie Jungles, Gunseli Onder, Jalal Samhoun, Balázs Győrffy, Karin M. Hardiman

**Affiliations:** ^1^ Department of Molecular and Integrative Physiology, University of Michigan, Ann Arbor, MI, USA; ^2^ Saint Mary's College, Notre Dame, IN, USA; ^3^ MTA TTK Lendület Cancer Biomarker Research Group, MTA-SE Pediatrics and Nephrology Research Group, Semmelweis University 2nd Department of Pediatrics, Budapest, Hungary; ^4^ Department of Surgery, University of Michigan, Ann Arbor, MI, USA

**Keywords:** hypoxia-inducible factor-3α, colorectal cancer, Janus kinase, signal transducer, activator of transcription 3

## Abstract

Hypoxic environment is critical in colorectal cancer (CRC) development. Most studies have mainly focused on hypoxia-inducible factor (HIF)-1α and HIF-2α as the major hypoxic transcription factors in CRC development and progression. However, the role of HIF-3α in CRC is not clear. Here we found that HIF-3α protein was increased in colorectal tumors from both mouse models and human patients. Moreover, increased HIF-3α expression was correlated with decreased survival. Overexpression of a long isoform of HIF-3α, HIF-3α1, increased cell growth in two CRC cell lines. Surprisingly, overexpressed HIF-3α1 was localized to the cytosol and increased phosphorylated signal transducer and activator of transcription 3 (p-STAT3). STAT3 inhibition effectively reduced p-STAT3 levels and cell growth induced by HIF-3α1. The activation of p-STAT3 was independent of the transcriptional activity of HIF-3α1. However, the inhibition of the upstream regulator Janus kinase (JAK) abolished HIF-3α1-induced p-STAT3 and cell growth. Together, these results demonstrated that HIF-3α1 promotes CRC cell growth by activation of the JAK-STAT3 signaling pathway through non-canonical transcription-independent mechanisms.

## INTRODUCTION

Hypoxia-inducible factors (HIFs) are transcription factors that mediate hypoxia signaling, which is crucial in many cellular process including cancer development and progression. HIF is a heterodimer consisting of a hypoxia-inducible alpha subunit (HIF-α) and a constitutively expressed beta subunit (HIF-1β, or aryl hydrocarbon receptor nuclear translocator [Arnt]) [1]. Under normoxic cell conditions, HIF-α is hydroxylated by the oxygen-sensitive prolyl hydroxylase domain protein (PHD) and recognized by the von Hippel-Lindau tumor suppressor protein (VHL) coupled to the E3 ubiquitin ligase complex to initiate its degradation [2, 3]. However, in a hypoxic environment, the binding of VHL to HIF-α is decreased, which results in an accumulation of HIF-α and activation of its target gene expression [4]. There are three major isomers of HIF-α: HIF-1α, HIF-2α, and HIF-3α. While numerous studies have shown that HIF-1α and HIF-2α are activated in both physiologic and pathologic conditions [5], very little is known about HIF-3α.

Unlike HIF-1α and HIF-2α, HIF-3α contains at least 10 predicted mRNA variants in humans from the utilization of different promoters, different transcription initiation sites, and alternative splicing have been identified [6]. HIF-1α and HIF-2α share a high homology and both have two prolyl sites and two transactivation domains (TADs, N-terminal and C-terminal), whereas HIF-3α has only one prolyl site and the N-terminal TAD [7]. Thus HIF-3α can still be induced by hypoxia [8], and certain long isoforms, such as HIF-3α1, can increase a distinct set of HIF-α target genes [7, 9]. Both HIF-1α and HIF-2α are overexpressed in colorectal cancer (CRC) tissues from patients [10, 11]. HIF-1α is required for pro-inflammatory regulation and promotes the survival of CRC cell lines under hypoxic condition [12, 13], whereas HIF-2α increases genes important in proinflammatory response, tumor growth and invasion in mice [14–17]. Here, we hypothesize that HIF-3α also promotes the development and progression of CRC.

This study demonstrates that HIF-3α is overexpressed in both mouse and human colorectal tumors, and predicts poor prognosis. Overexpression of HIF-3α1 greatly increases colorectal tumor cell growth. Interestingly, overexpressed HIF-3α1 is localized to the cytosol and can strongly activate the pro-survival signal transducer and activator of transcription 3 (STAT3) signaling, which is essential for HIF-3α-promoted CRC cell growth. Further investigation shows that the upstream signal Janus kinase (JAK) is important for HIF-3α-promoted STAT3 activation. Together, these data uncovered a novel role for HIF-3α in activating the JAK-STAT3 signaling cascade to influence the cell proliferation and growth of CRC.

## RESULTS

### HIF-3α is overexpressed in both mouse and human colorectal tumors and predicts poor prognosis

**Figure 1:**
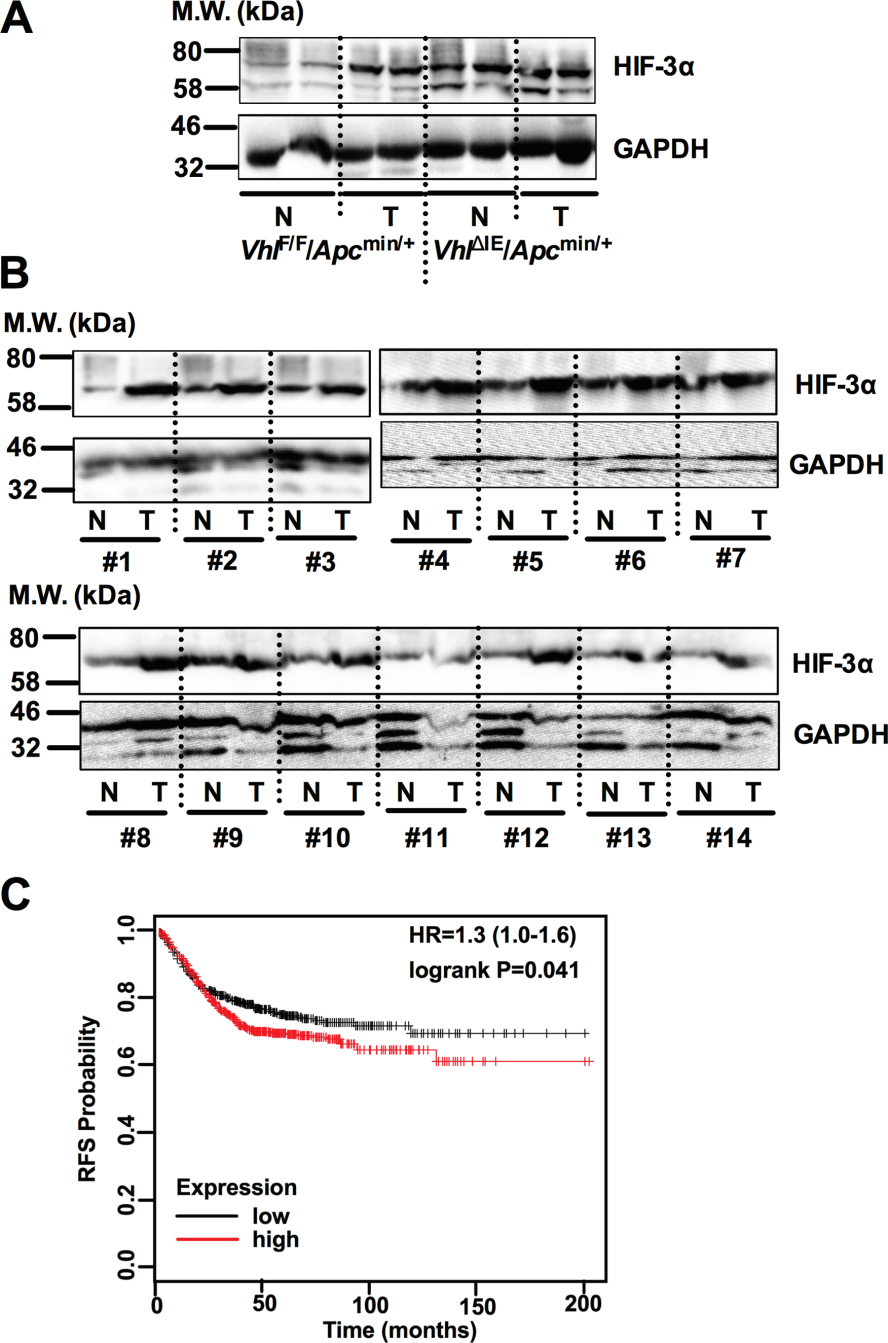
HIF-3α is overexpressed in CRC Western blot analysis for HIF-3α and GAPDH from normal (N) and tumor (T) colorectal tissues of (**A**) 3-month-old *Vhl*^F/F^/*Apc*^min/+^, and *Vhl*^ΔIE^/*Apc*^min/+^ mice or (**B**) 14 pairs of human colorectal tumor. (**C**) Kaplan-Meier survival curves of HIF-3α gene in colorectal tumor patients. M.W., molecular weight.

Previously, we have reported that intestine-specific disruption of *Vhl* (*Vhl^ΔIE^*) activates HIF signaling, and when these mice are crossed to the *Apc*^min/+^ intestinal tumor model (*Vhl^ΔIE^/Apc^min/+^*), colorectal tumorigenesis is robustly increased compared with littermate control mice (*Vhl^F/F^/Apc^min/+^*) [14]. HIF-3α expression was further assessed in the normal and tumor tissues of *Vhl^ΔIE^/Apc^min/+^* and *Vhl^F/F^/Apc^min/+^* mice (Figure 1A). Tumors isolated from *Vhl^F/F^/Apc^min/+^* mice demonstrate an increase in HIF-3α expression compared to their adjacent normal tissue. Furthermore, the *Vhl* knockout mouse model (*Vhl^ΔIE^/Apc^min/+^*) demonstrates an increase in HIF-3α expression in both normal and tumor tissue samples. In addition, colorectal tumors isolated from patients demonstrated an increase in HIF-3α compared to their adjacent normal tissue (Figure 1B). These data demonstrate that HIF-3α is overexpressed in both mouse and human colorectal tumors. Moreover, Kaplan-Meier survival curves were generated and stratified using datasets published under the GEO accession numbers GSE12945, GSE14333, GSE17538, GSE31595, GSE33114, GSE37892, GSE39582, and GSE41258. Increased expression of HIF-3α in patient specimens predicted worse patient survival (Figure 1C). Together, these data suggest that HIF-3α is important in colorectal tumor development and cancer progression.

### Overexpression of HIF-3α1 in CRC cells promotes cell growth

**Figure 2:**
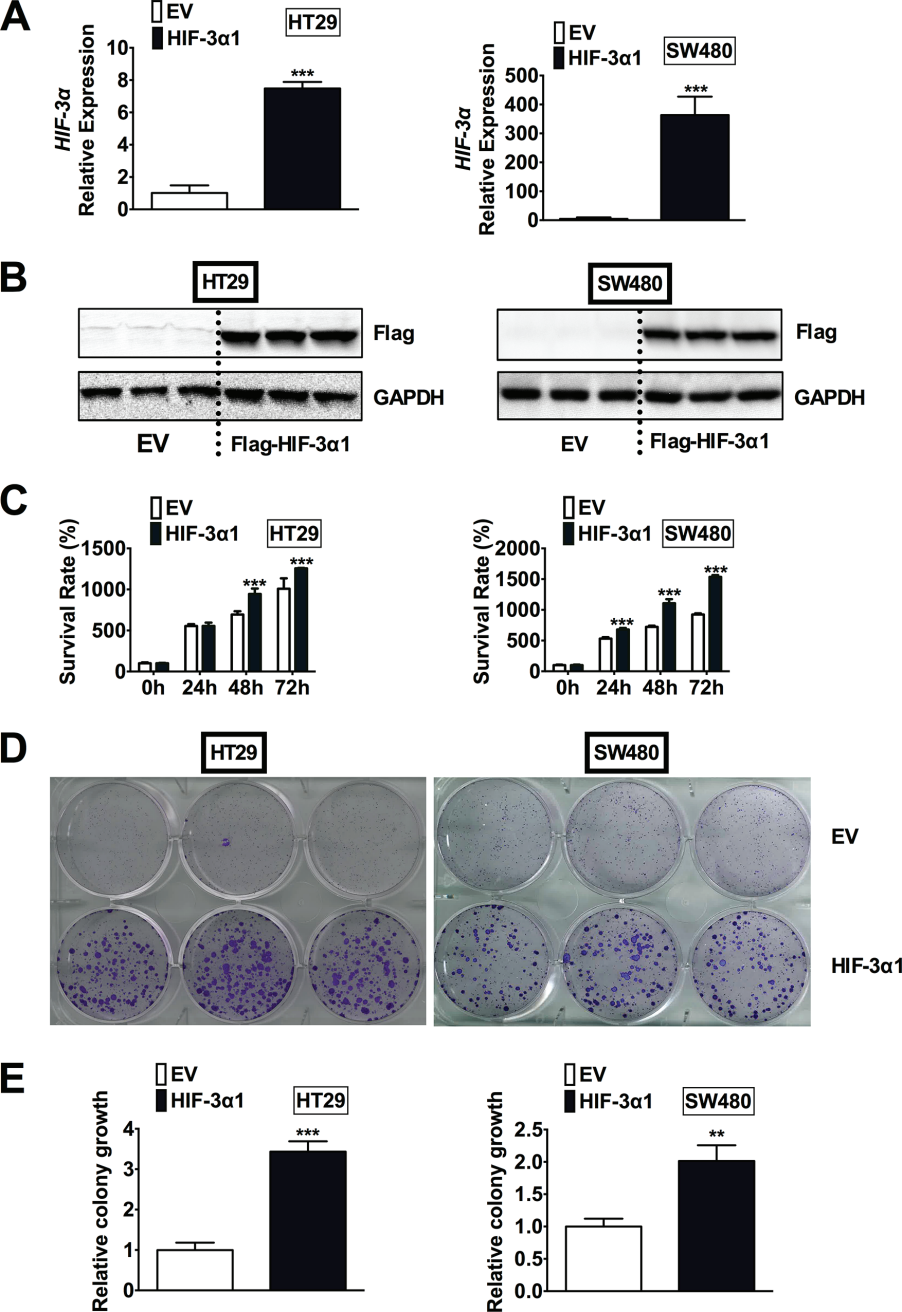
Overexpression of HIF-3α1 enhances CRC cell and colony growth (**A**) qPCR analysis of HIF-3α gene expression, (**B**) Western blot analysis of Flag protein, (**C**) cell survival determined by MTT assay, (**D**) colony formation detected by crystal violet assay and (**E**) quantification of colonies formed in flag-tagged HIF-3α1-overexpressing or empty vector (EV) lentivirus infected HT29 and SW480 CRC cells. **p < 0.01, ***p < 0.001 compared with EV.

To determine if the increased HIF-3α expression contributes to the increase in colorectal tumorigenesis, HIF-3α1 was stably overexpressed in two CRC-derived cell lines, HT29 and SW480. We chose to overexpress HIF-3α1 since it is the longer isoform of human HIF-3α and the size of HIF-3α1 was close to the increased HIF-3α detected in human CRC tumors by Western blot analysis (Figure 1B). In addition, HIF-3α1 has a significantly higher activity than the shorter isoforms such as HIF-3α4, which has no TAD and thus no activity [9]. By qPCR analysis, the HIF-3α mRNA expression was significantly increased in the lentiviral HIF-3α1 infected cells compared to the lentiviral empty vector (EV) infected cells in both HT29 and SW480 cells (Figure 2A). Western blot analysis for flag-tagged HIF-3α1 confirmed stable overexpression of HIF-3α1 (Figure 2B). Cell growth was assessed and the results indicated that the HIF-3α1 overexpressing cells exhibited increased growth rate over a 72-hour period compared to EV cells (Figure 2C). A colony formation assay was performed to confirm these results, and as expected, the HIF-3α1 overexpressing cells formed more colonies compared to the EV cells (Figure 2D and 2E). These results demonstrate that HIF-3α1 overexpression may contribute to colorectal tumorigenesis and cancer progression.

### HIF-3α1 is localized in the cytosol in CRC-derived cell lines and in the colon in mouse models

**Figure 3:**
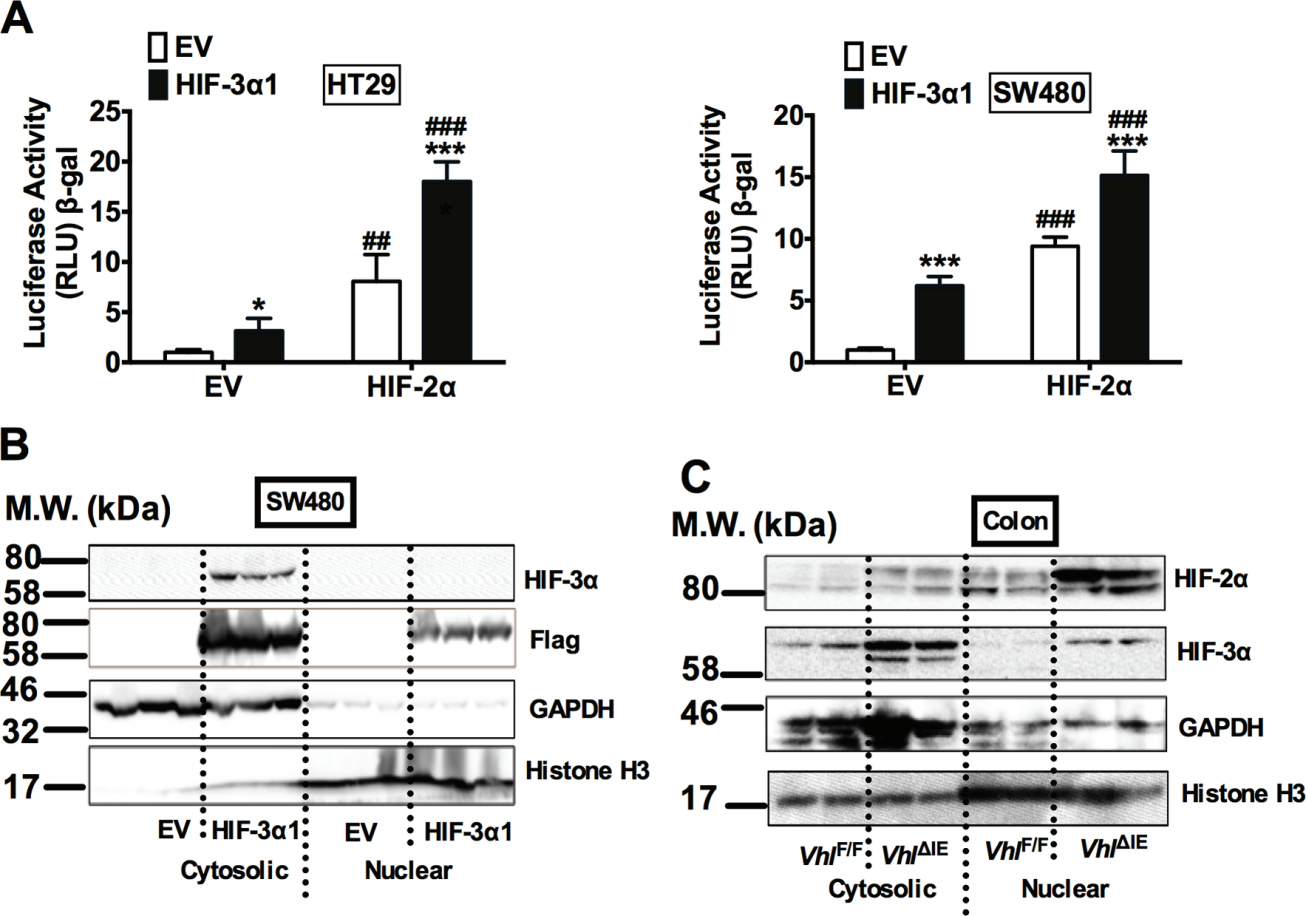
HIF-3α1 can activates hypoxia response gene in CRC cells and is majorly located in the cytosol when stabilized (**A**) Enolase promoter (P2.1) luciferase assay in HIF-3α1-overexpressing or EV lentivirus infected HT29 and SW480 CRC cells. Cells were transfected with HIF-2α or EV plasmids. (**B**) Western blot analysis in the cytosolic and nuclear fraction from HIF-3α1-overexpressing or EV lentivirus infected SW480 CRC cells or colorectal tissues of *Vhl*^F/F^ and *Vhl*^ΔIE^ mice. *p < 0.05, ***p < 0.001 compared with EV control cell line. ^##^*p* < 0.01, ^###^*p* < 0.001 compared with EV control plasmids. M.W., molecular weight.

To test whether HIF-3α1 increases cell growth through regulating canonical hypoxia response genes, the luciferase assay for canonical hypoxia target gene Enolase promoter (P2.1) was examined. HIF-3α1 overexpression increased the P2.1 luciferase activity, and this was further potentiated by HIF-2α (Figure 3A). This suggests that HIF-3α1 has a transcriptional activity. To confirm this, the cellular distribution of HIF-3α1 was examined. Surprisingly, though Flag antibody can recognize both nuclear and cytosol flag-tagged HIF-3α1 by Western blot analysis, the HIF-3α antibody can only detect HIF-3α1 in the cytosol fraction in the SW480 cells (Figure 3B). Consistent with this *in vitro* cell line data, the majority of HIF-3α protein was found to be located in the cytosol fraction from colon extracts of *Vhl^ΔIE^* mice, whereas the majority of HIF-2α protein was in the nuclear fraction (Figure 3C). These data suggest that HIF-3α1 increased CRC cell growth may not through its transcriptional activity.

### Overexpression of HIF-3α1 activates STAT3 signaling

**Figure 4:**
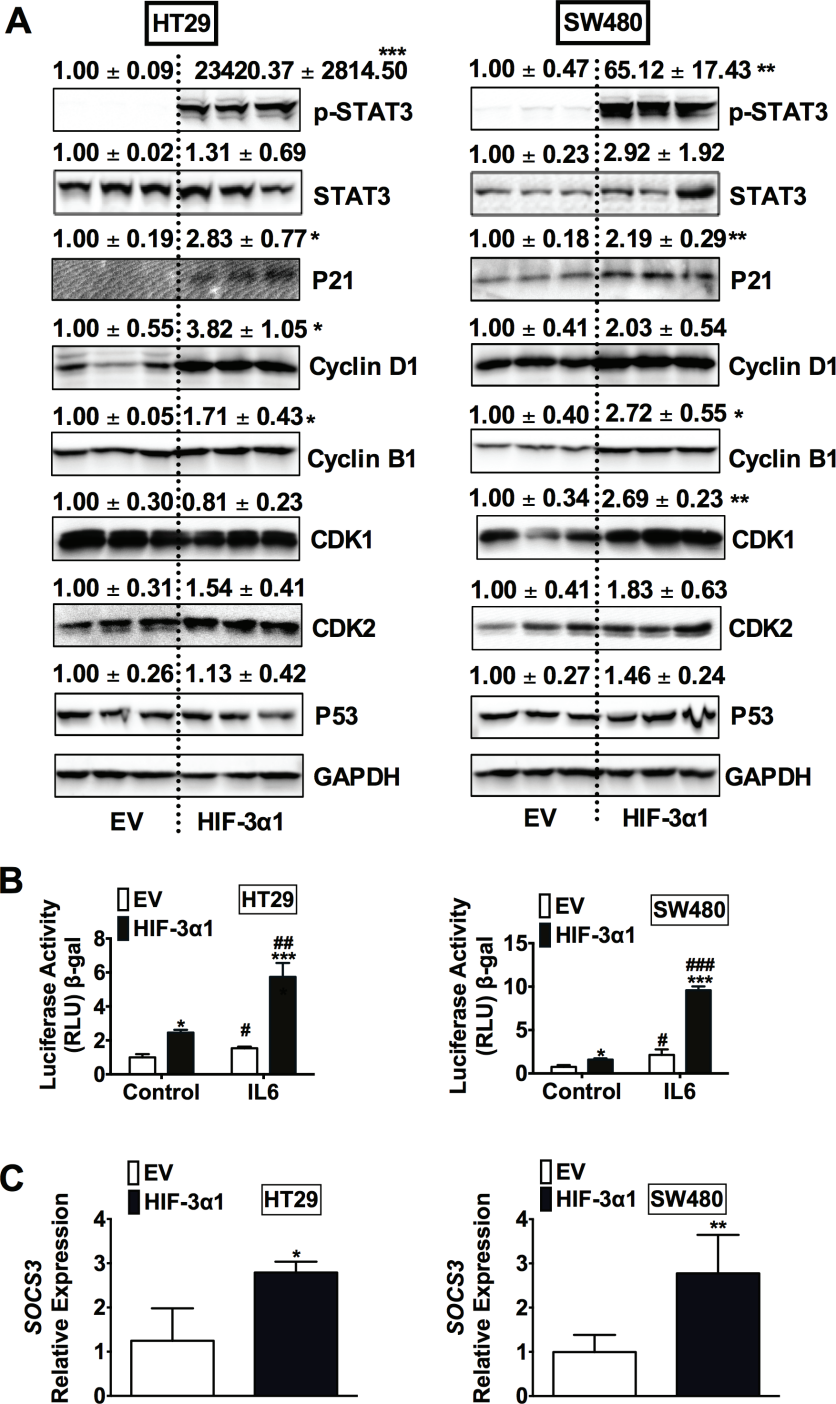
Overexpression of HIF-3α1 activates STAT3 signaling in CRC cells (**A**) Western blot analysis in whole cell extracts from HIF-3α1-overexpressing or EV lentivirus-infected HT29 or SW480 CRC cells. (**B**) STAT3 activity luciferase assay in HIF-3α1-overexpressing or EV lentivirus infected HT29 and SW480 CRC cells. Cells were treated with or without IL6 (10 ng/mL) for 24 hours. (**C**) qPCR analysis of SOCS3 gene expression in HIF-3α1-overexpressing or EV lentivirus infected HT29 and SW480 CRC cells. *p < 0.05, **p < 0.01, ***p < 0.001 compared with EV control cell line. ^##^*p* < 0.01, ^###^*p* < 0.001 compared with untreated controls.

To determine the mechanisms responsible for HIF-3α1-enhanced cell growth, Western blot analysis was performed for cell cycle, cell survival and apoptosis (Figure 4A). A robust increase in phosphorylated signal transducer and activator of transcription 3 (p-STAT3) was observed in HIF-3α1 overexpressing cells compared to EV. STAT3 is a protein known to be important in cell proliferation and cell survival in CRC, which is primarily activated by interleukin-6 (IL-6) signaling. Consistent with an increase in p-STAT3, STAT3 activity was also increased in HIF-3α1 overexpressing cell lines, and the activity was further enhanced by IL6 stimulation (Figure 4B). Furthermore, the gene expression of *SOCS3*, a known target gene of STAT3 signaling, was increased by HIF-3α1 overexpression (Figure 4C). Together, these data indicate that HIF-3α can robustly activate STAT3 signaling.

### STAT3 inhibition decreases HIF-3α1-promoted cell growth

**Figure 5:**
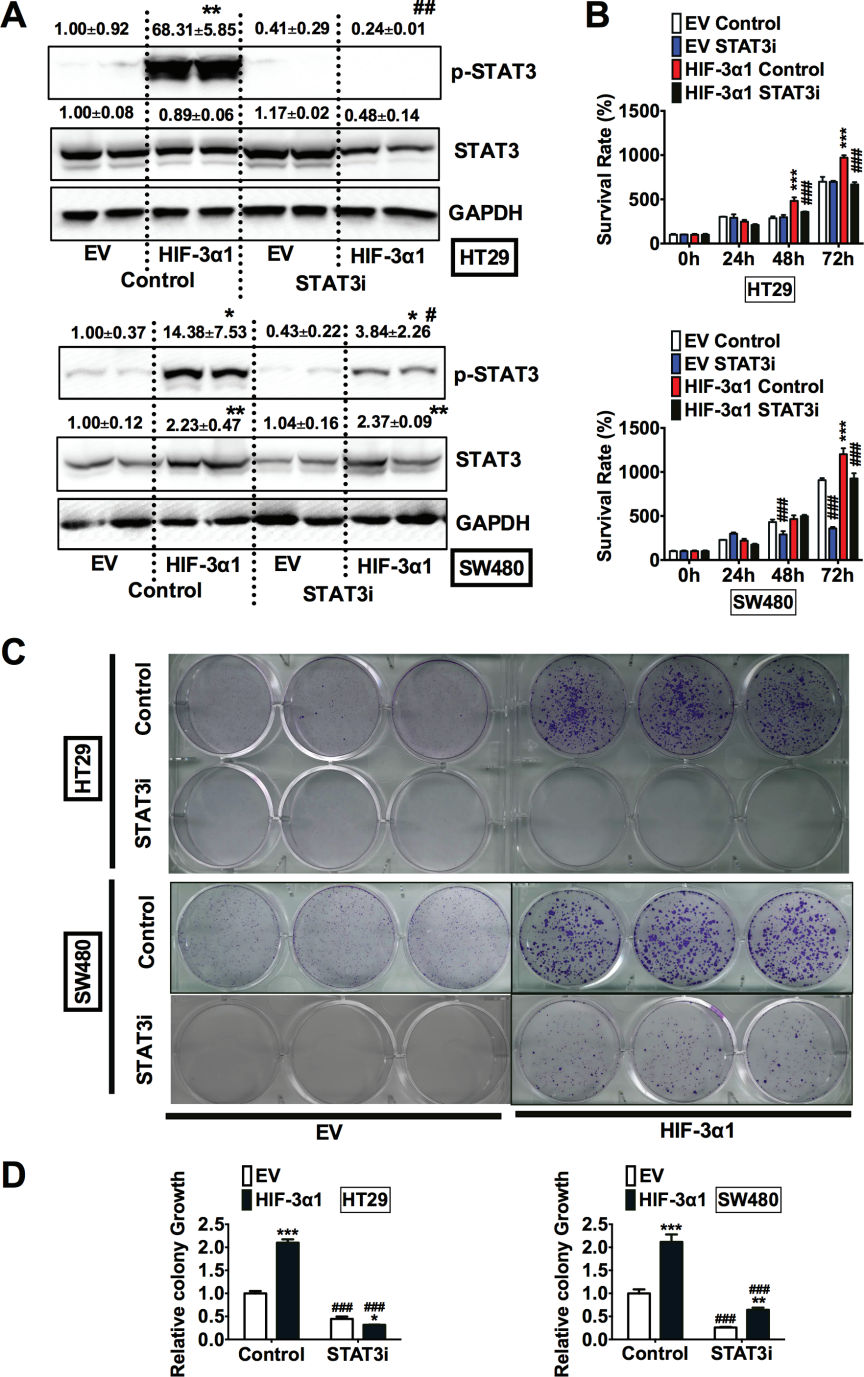
Inhibition of STAT3 decreases HIF-3α1-enhanced CRC cell and colony growth (**A**) Western blot analysis of p-STAT3 and STAT3 in whole cell extracts, (**B**) cell survival determined by MTT assay, (**C**) colony formation detected by crystal violet assay and (**D**) quantification of colonies formed from STAT3 inhibitor (STAT3i) treated or untreated HIF-3α1-overexpressing or EV lentivirus-infected HT29 or SW480 CRC cells. *p < 0.05, **p < 0.01, ***p < 0.001 compared with EV control cell line. ^#^*p* < 0.05, ^##^*p* < 0.01, ^###^*p* < 0.001 compared with untreated controls.

To confirm the critical role of STAT3 in HIF-3α1-promoted cell growth, HT29 and SW480 EV and HIF-3α1 cells were treated with S3I-201, a STAT3 inhibitor (STAT3i). The specificity of this STAT3i is demonstrated by the fact that it reduced JAK1 increased STAT3 activity (Figure S1A), but not HIF-1α induced P2.1 luciferase activity (Figure S1B). Western blot analysis confirmed that the STAT3i successfully reduced the HIF-3α1 increased p-STAT3 levels in both HT29 and SW480 cells (Figure 5A). STAT3 inhibition resulted in decreased growth in HIF-3α1-overexpressing cells, whereas STAT3i did not result in significant decrease in EV cells. (Figure 5B). To confirm these results, a colony formation assay was performed to assess the relative growth of the cells treated with STAT3i compared to the untreated cells (Figure 5C and 5D). The results of the colony formation assay showed that STAT3i significantly reduced HIF-3α1-enhanced colony growth. Together, these data indicate that STAT3 activation is necessary for HIF-3α1-promoted cell proliferation.

### HIF-3α-promoted activation of STAT3 is independent of its transcriptional activity

**Figure 6:**
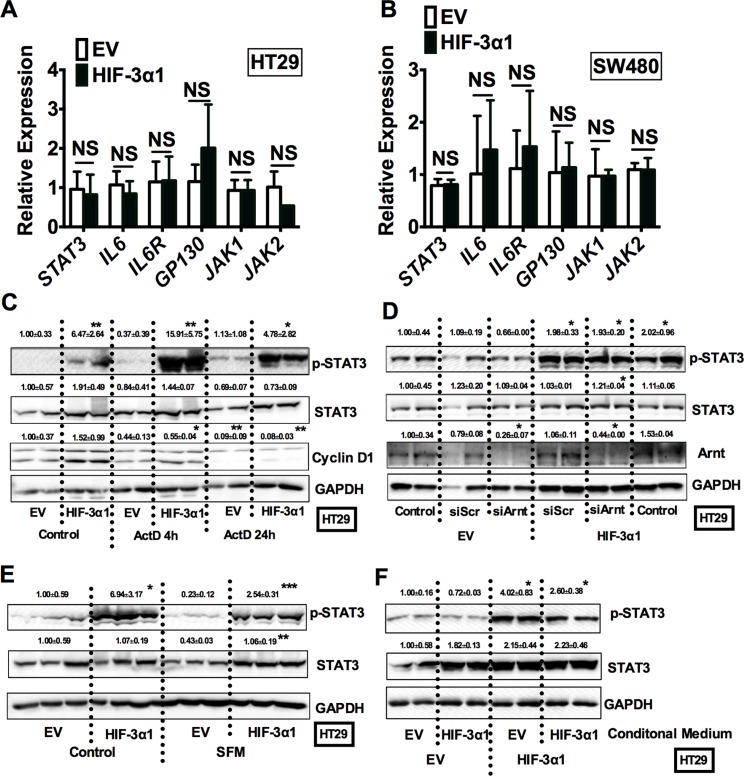
HIF-3α1-promoted activation of STAT3 is not through increased transcription qPCR analysis in HIF-3α1-overexpressing or EV lentivirus infected (**A**) HT29 and (**B**) SW480 CRC cells. Western blot analysis of p-STAT3 and STAT3 in whole cell extracts from (**C**) transcription inhibitor Actinomycin D (ActD), (**D**) siArnt, siScr treated or untreated control, (**E**) serum free medium (SFM) treated or untreated, or (**F**) conditional medium collected from cell culture treated HIF-3α1-overexpressing or EV lentivirus-infected HT29 or SW480 CRC cells. *p < 0.05, **p < 0.01, ***p < 0.001 compared with EV control cell line. NS, not significant.

To further understand how HIF-3α1 induces STAT3 activation, mRNA analysis for the STAT3 signaling pathways was assessed. Real-time qPCR analysis showed that the *STAT3* mRNA levels were not changed by overexpression of HIF-3α1 (Figure 6A and 6B). Furthermore, several genes such as IL6, IL6R and GP130 that are important in STAT3 activation were not increased by overexpression of HIF-3α1 either. HIF transcription factors recently have been shown to have non-transcriptional function important in cell cycle and cancer progression [18, 19]. Under hypoxia, cells switch to selective cap-dependent translation initiation machinery for protein synthesis [20]. However this has not been shown for HIF-3α1. To understand if transcriptional activation by HIF-3α1 was required for the enhanced STAT3 activation, cells were treated with Actinomycin D (Act D), a transcription inhibitor. Act D time-dependently decreased the expression of cyclin D1, which indicates the effectiveness of this compound. However, Act D did not inhibit the p-STAT3 activation by HIF-3α1 (Figure 6C). Furthermore, knocking down Arnt, a cofactor essential for the transcriptional activity of all isoforms of HIF-α, effectively reduces the protein levels of Arnt to about 30%–40% compared to scrambled control in both EV and HIF-3α overexpressing cell lines, but it did not reduce the HIF-3α-increased p-STAT3 level (Figure 6D). These results suggest that HIF-3α1 activated p-STAT3 is via a non-transcriptional mechanism. Since STAT3 can be activated by several growth factors such as EGF [21], to exclude the effects of serum containing factors, cells were incubated in serum-free medium (SFM) (Figure 6E). The p-STAT3 level was slightly decreased but still significantly increased compared to EV cells. To further evaluate if paracrine-signaling factors led to an increase in p-STAT3 by HIF-3α1, EV cells were treated with conditioned media from HIF-3α1 overexpressing cells. HIF-3α1 conditioned media did not activate p-STAT3 in EV cells (Figure 6F), suggesting that the activation of STAT3 is a cell intrinsic mechanism.

### HIF-3α-activated STAT3 requires JAK

**Figure 7:**
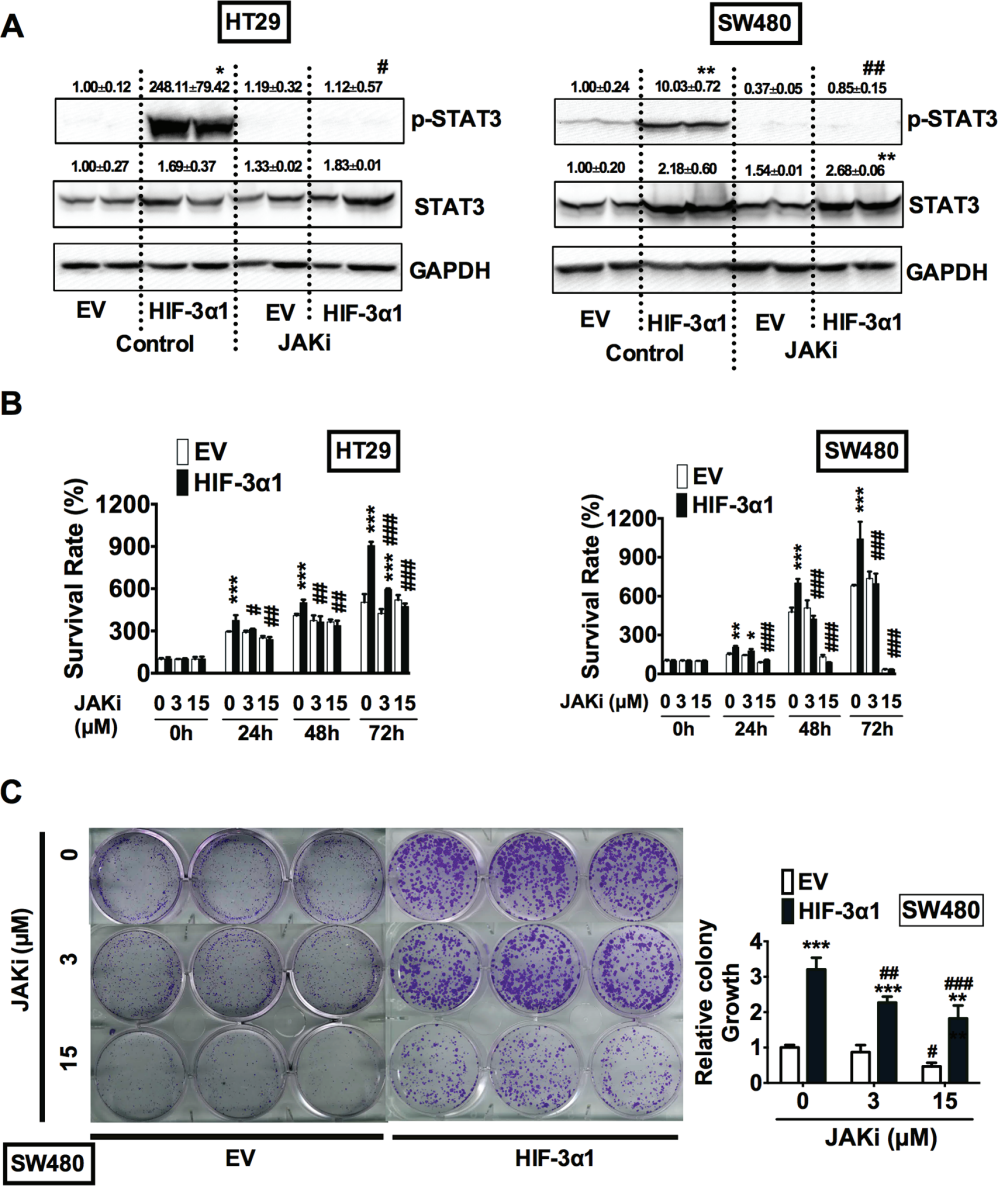
HIF-3α-promoted activation of STAT3 requires JAK signaling (**A**) Western blot analysis of p-STAT3 and STAT3 in whole cell extracts, (**B**) cell survival determined by MTT assay, (**C**) colony formation detected by crystal violet assay and quantification of colonies formed from JAK1/2 inhibitor (JAKi) treated or untreated HIF3A1-overexpressing or EV lentivirus-infected HT29 or SW480 CRC cells. *p < 0.05, **p < 0.01, ***p < 0.001 compared with EV control cell line. ^#^*p* < 0.05, ^##^*p* < 0.01, ^###^*p* < 0.001 compared with untreated controls.

JAK is a known upstream kinase that phosphorylates STAT3, which allows STAT3 to translocate into the nucleus and initiate transcription [22–24]. To determine whether HIF-3α1 is capable of binding with JAK and triggering the JAK-STAT signaling cascade, cells were treated with Ruxolitinib, a JAK1/2 inhibitor (JAKi). Western Blot analysis showed that JAK inhibition completely abolished the HIF-3α1-increased STAT3 activation (Figure 7A). MTT assay indicated that JAKi abrogated HIF-3α1-increased growth (Figure 7B). Colony formation assay further confirmed that JAKi reduced colony growth of HIF-3α-overexpressing and EV cells in a dose-dependent manner (Figure 7C and 7D). Together, these data indicate that HIF-3α1 increases CRC cell proliferation and survival by activation of the JAK-STAT signaling pathway

## DISCUSSION

Hypoxia is a hallmark of solid tumors. Through increasing the stability of HIF-α, hypoxia can activate the expression of numerous genes involved in cell metabolism, cell survival, cell proliferation and cell apoptosis. In the intestine, activation of HIF-1α does not increase tumorigenesis in mice [25], whereas HIF-2α promotes the development of CRC [14]. Here, we demonstrate that HIF-3α1, also promotes CRC cell growth. Interestingly, the mechanism was not through transcriptional regulation, but by activation of the JAK-STAT3 signaling.

The JAK-STAT3 signaling is aberrantly activated in the CRC tissues and is critical in promoting carcinogenesis [26, 27]. Phosphorylation of STAT3 is positively correlated with the tumor invasion of colorectal adenocarcinoma in human [28]. Excess activation of STAT3 in enterocytes promotes, whereas ablation of STAT3 reduces tumor cell proliferation through G1 and G2/M cell cycle progression in mouse models of colitis-associated CRC [29]. STAT3 is activated by many growth factors through their receptor tyrosine kinase activity or cytokines such as IL-6 through JAK family kinases. Pharmacological inhibition of JAK activity inhibits progression of gastrointestinal tumors in mice [27]. The current study demonstrates that JAK-STAT3 signaling is also activated by HIF-3α1, which provides a novel insight in JAK-STAT3 activity modulation.

HIF-3α is originally identified to have a repressive activity since its short isoforms such as HIF-3α4 inhibit the action of HIF-1α and HIF-2α [30]. However, a recent report shows that HIF-3α1 also has a strong transactivation activity [9]. We also found that HIF-3α1 can potentiate the activity of HIF-2α in CRC cells. Interestingly, HIF-3α1 can also stimulate cellular JAK-STAT3 activity through a transcription-independent mechanism. There are at least three major isoforms of JAK: JAK1, JAK2 and JAK3. Western blot analysis failed to detect the activation of phospho-JAK2 in HIF-3α1 overexpression cells, further studies are still needed to understand which isoform is responsible for HIF-3α-increased STAT3 activation and to dissect the precise mechanisms for how HIF-3α binds with JAK. Also, it is necessary to validate our results *in vivo* using mouse models of colorectal tumors.

In conclusion, our findings demonstrate that HIF-3α activates JAK-STAT3 signaling pathway to facilitate CRC cell growth. Thus, together with our previous report about the tumor-promoting role of HIF-2α in CRC, inhibiting hypoxia signaling may provide a promising strategy for the prevention and treatment of CRC.

## MATERIALS AND METHODS

### Animals

*Vhl*^F/F^, *Vhl*^ΔIE^, *Vhl*^F/F^/*Apc*^min/+^ and *Vhl*^ΔIE^/*Apc*^min^ mice were described previously [15]. All mice were on a 129S6/SvEv background and maintained in standard cages in a light and temperature-controlled room and were allowed standard chow and water *ad libitum*. All animal studies were carried out in accordance with the Institute of Laboratory Animal Resources guidelines and approved by the University Committee on the Use and Care of Animals at the University of Michigan.

### Human colorectal tumor tissues

Human colorectal tumor and adjacent normal tissues were obtained from individuals undergoing colorectal tumor removal surgery. The Institutional Review Board of the University of Michigan approved the use of these materials.

### Western blot analysis

Whole-cell or nuclear extracts were isolated as previously described [14]. Proteins were separated and transferred to nitrocellulose membranes using standard methods. Antibodies against HIF-3α (Abcam Inc., Cambridge, MA), Flag (Sigma, St. Louis, MO), HIF-2α (Novus Biologicals, Littleton, CO), Histone H3, p-STAT3, STAT3, P21, P53 (Cell Signaling Technology, Danvers, MA), Cyclin D1, Cyclin B1, CDK1, CDK2, Arnt and GAPDH (Santa Cruz Biotechnology, Dallas, TX) were used.

### Meta-analysis of CRC samples

CRC gene expression datasets with survival were identified in GEO using the search keywords “colorectal”, “cancer”, and “microarray” (http://www.ncbi.nlm.nih.gov/geo/). Only publications providing raw data, clinical survival information, and containing at least 30 patients were included. The gene chips were MAS 5.0 normalized in the R statistical environment (http://www.R-project.org) using the Bioconductor package Affy (http://www.bioconductor.org). Survival analysis using Cox proportional hazards regression was performed as described previously [31]. The most reliable probe sets for each gene were selected using Jetset. All percentiles between lower and upper quartiles are computed, and the best performing threshold is used as a cutoff. The values lower than the cutoff are designated as “low”, whereas the values higher than cutoff are designated as “high”.

### Cell culture

HT29 and SW480 CRC cells were obtained from ATCC and maintained at 37°C in 5% CO2 and 21% O2. Cells were cultured in Dulbecco's modified eagle medium (DMEM) supplemented with 10% Fetal Bovine Serum (FBS) and 1% antibiotic/antimycotic. Early-passage (P10) stable HIF3A1-expressing and parental HT29 or SW480 cells were generated by lentiviral infection of pLentilox-puro-HIF3A1 or pLentilox-puro empty vector (EV) and selected by 2-μg/mL puromycin. The cells were maintained in growth media as described above albeit supplemented with 1 μg/mL of puromycin. For condition medium collection, fresh FBS-free culture medium DMEM was added to the 10 cm plates with cells and incubated overnight, the next day the culture medium was collected and centrifuged at full speed for 10 min to get rid of floating cell debris. One mL of the supernatant was added to the cells plated in 6-well plates the day before as indicated.

### Quantitative real-time RT-PCR (qPCR)

RNA was isolated from frozen tissue using Isol-RNA lysis reagent (3 Prime, Gaithersburg, MD) and quantitated using the NanoDrop 2000 (NanoDrop products, Wilmington, DE). RNA with a purity (260/280 ratio) of approximately 2.0 was reverse-transcribed using M-MLV Reverse Transcriptase (Fisher Scientific, Waltham, MD). mRNA expression was measured by Real Time RT-PCR using SYBR green (Life Technologies, Carlsbad, CA) (primers are listed in Supplementary Table 1). Ct values were normalized to β-actin and expressed as fold difference from controls.

### MTT assay

Cells were plated at a concentration of 5 × 10^4^ cells/mL in a 24-well plate. After 24-, 48-, or 72-hr culture in the presence or absence of STAT3 inhibitor (STAT3i, 100 μM), JAK1/2 inhibitor (JAKi, 3 or 15 μM), 125 μL 5 mg/mL Thiazolyl Blue Tetrazolium Bromide (MTT, Sigma, MO) was added to each well and incubated for 30 min. Dimethyl sulfoxide was added and absorbance was measured at 570 nm.

### Colony formation assay

Cells were plated at a concentration of 500 cells/mL in a 6-well plate. Cells were treated with or without STAT3i (100 μM) or JAKi (3 or 15 μM) every two days for 10 days. Formed colonies were fixed with 10% neutral buffered formalin solution and stained with 0.01% crystal violet for 30 minutes. Excess crystal violet was washed with Millipore H_2_O for 3 times and allowed to dry. Digital images of the colonies were obtained. Methanol was added to dissolve the crystal violet and absorbance at 540 nm was measured.

### Luciferase assay

Cells were seeded into a 24-well plate at a cell density of 5 × 10^4^ cells per well. Enolase promoter luciferase reporter constructs P2.1 or STAT3 activity reporter luciferase construct pGL4.47 [luc2P/SIE/Hygro] was co-transfected with HIF-1α, HIF-2α, JAK1 or empty vector (EV) into cells with polyethylenimine (PEI; Polysciences Inc., Warrington, PA). Cells were treated with 10 ng/mL IL6 or 100 μM STAT3i at 24 hours after transfection as indicated. Cells were lysed in reporter lysis buffer (Promega, Madison, WI), and firefly luciferase activity was measured and normalized to β-galactosidase (β-gal) activity 48 hours after transfection.

### siRNA knockdown assay

Cells were seeded into a 12-well plate at a cell density of 5 × 10^4^ cells per well. Lipofectamine 2000 (Life Technologies, Carlsbad, CA) was used for transfection of 50 nM Arnt siRNA (siArnt) or scrambled siRNA (siScr, GE Dharmacon, Lafayette, CO). Cells were collected at 48 hours after transfection for Western blot analysis as described above.

### Statistical analysis

Results are expressed as mean ± S.D. Western blot analysis were quantified with Image J. *p* values were calculated by independent *t*-test and two-way ANOVA. *p* < 0.05 was considered significant.

## SUPPLEMENTARY MATERIALS TABLE AND FIGURE



## References

[1] Semenza GL, Wang GL (1992). A nuclear factor induced by hypoxia via de novo protein synthesis binds to the human erythropoietin gene enhancer at a site required for transcriptional activation. Mol Cell Biol.

[2] Ivan M, Kondo K, Yang H, Kim W, Valiando J, Ohh M, Salic A, Asara JM, Lane WS, Kaelin WG (2001). HIFalpha targeted for VHL-mediated destruction by proline hydroxylation: implications for O2 sensing. Science.

[3] Jaakkola P, Mole DR, Tian YM, Wilson MI, Gielbert J, Gaskell SJ, von Kriegsheim A, Hebestreit HF, Mukherji M, Schofield CJ, Maxwell PH, Pugh CW, Ratcliffe PJ (2001). Targeting of HIF-alpha to the von Hippel-Lindau ubiquitylation complex by O2-regulated prolyl hydroxylation. Science.

[4] Wang GL, Semenza GL (1993). General involvement of hypoxia-inducible factor 1 in transcriptional response to hypoxia. Proc Natl Acad Sci U S A.

[5] Loboda A, Jozkowicz A, Dulak J (2010). HIF-1 and HIF-2 transcription factors—similar but not identical. Mol Cells.

[6] Pasanen A, Heikkila M, Rautavuoma K, Hirsila M, Kivirikko KI, Myllyharju J (2010). Hypoxia-inducible factor (HIF)-3 alpha is subject to extensive alternative splicing in human tissues and cancer cells and is regulated by HIF-1 but not HIF-2. Int J Biochem Cell Biol.

[7] Heikkila M, Pasanen A, Kivirikko KI, Myllyharju J (2011). Roles of the human hypoxia-inducible factor (HIF)-3alpha variants in the hypoxia response. Cell Mol Life Sci.

[8] Li QF, Wang XR, Yang YW, Lin H (2006). Hypoxia upregulates hypoxia inducible factor (HIF)-3alpha expression in lung epithelial cells: characterization and comparison with HIF-1 alpha. Cell Res.

[9] Zhang P, Yao Q, Lu L, Li Y, Chen PJ, Duan C (2014). Hypoxia-inducible factor 3 is an oxygen-dependent transcription activator and regulates a distinct transcriptional response to hypoxia. Cell Rep.

[10] Talks KL, Turley H, Gatter KC, Maxwell PH, Pugh CW, Ratcliffe PJ, Harris AL (2000). The expression and distribution of the hypoxia-inducible factors HIF-1alpha and HIF-2alpha in normal human tissues, cancers, and tumor-associated macrophages. Am J Pathol.

[11] Zhong H, De Marzo AM, Laughner E, Lim M, Hilton DA, Zagzag D, Buechler P, Isaacs WB, Semenza GL, Simons JW (1999). Overexpression of hypoxia-inducible factor 1alpha in common human cancers and their metastases. Cancer Res.

[12] Kaidi A, Qualtrough D, Williams AC, Paraskeva C (2006). Direct transcriptional up-regulation of cyclooxygenase-2 by hypoxia-inducible factor (HIF)-1 promotes colorectal tumor cell survival and enhances HIF-1 transcriptional activity during hypoxia. Cancer Res.

[13] Muller-Edenborn K, Leger K, Glaus Garzon JF, Oertli C, Mirsaidi A, Richards PJ, Rehrauer H, Spielmann P, Hoogewijs D, Borsig L, Hottiger MO, Wenger RH (2015). Hypoxia attenuates the proinflammatory response in colon cancer cells by regulating IkappaB. Oncotarget.

[14] Xue X, Taylor M, Anderson E, Hao C, Qu A, Greenson JK, Zimmermann EM, Gonzalez FJ, Shah YM (2012). Hypoxia-inducible factor-2alpha activation promotes colorectal cancer progression by dysregulating iron homeostasis. Cancer Res.

[15] Xue X, Shah YM (2013). Hypoxia-inducible factor-2alpha is essential in activating the COX2/mPGES-1/PGE2 signaling axis in colon cancer. Carcinogenesis.

[16] Xue X, Ramakrishnan S, Anderson E, Taylor M, Zimmermann EM, Spence JR, Huang S, Greenson JK, Shah YM (2013). Endothelial PAS domain protein 1 activates the inflammatory response in the intestinal epithelium to promote colitis in mice. Gastroenterology.

[17] Evensen NA, Li Y, Kuscu C, Liu J, Cathcart J, Banach A, Zhang Q, Li E, Joshi S, Yang J, Denoya PI, Pastorekova S, Zucker S (2015). Hypoxia promotes colon cancer dissemination through up-regulation of cell migration-inducing protein (CEMIP). Oncotarget.

[18] Hubbi ME, Kshitiz, Gilkes DM, Rey S, Wong CC, Luo W, Kim DH, Dang CV, Levchenko A, Semenza GL (2013). A nontranscriptional role for HIF-1alpha as a direct inhibitor of DNA replication. Sci Signal.

[19] Villa JC, Chiu D, Brandes AH, Escorcia FE, Villa CH, Maguire WF, Hu CJ, de Stanchina E, Simon MC, Sisodia SS, Scheinberg DA, Li YM (2014). Nontranscriptional role of Hif-1 alpha in activation of gamma-secretase and notch signaling in breast cancer. Cell Rep.

[20] Uniacke J, Holterman CE, Lachance G, Franovic A, Jacob MD, Fabian MR, Payette J, Holcik M, Pause A, Lee S (2012). An oxygen-regulated switch in the protein synthesis machinery. Nature.

[21] Park OK, Schaefer TS, Nathans D (1996). *In vitro* activation of Stat3 by epidermal growth factor receptor kinase. Proc Natl Acad Sci U S A.

[22] Zhong Z, Wen Z, Darnell JE (1994). Stat3: a STAT family member activated by tyrosine phosphorylation in response to epidermal growth factor and interleukin-6. Science.

[23] O'Shea JJ, Gadina M, Schreiber RD (2002). Cytokine signaling in 2002: new surprises in the Jak/Stat pathway. Cell.

[24] O'Shea JJ, Holland SM, Staudt LM (2013). JAKs and STATs in immunity, immunodeficiency, and cancer. N Engl J Med.

[25] Xue X, Ramakrishnan SK, Shah YM (2014). Activation of HIF-1 alpha does not increase intestinal tumorigenesis. Am J Physiol Gastrointest Liver Physiol.

[26] Phesse TJ, Buchert M, Stuart E, Flanagan DJ, Faux M, Afshar-Sterle S, Walker F, Zhang HH, Nowell CJ, Jorissen R, Tan CW, Hirokawa Y, Eissmann MF (2014). Partial inhibition of gp130-Jak-Stat3 signaling prevents Wnt-beta-catenin-mediated intestinal tumor growth and regeneration. Sci Signal.

[27] Stuart E, Buchert M, Putoczki T, Thiem S, Farid R, Elzer J, Huszar D, Waring PM, Phesse TJ, Ernst M (2014). Therapeutic inhibition of Jak activity inhibits progression of gastrointestinal tumors in mice. Mol Cancer Ther.

[28] Kusaba T, Nakayama T, Yamazumi K, Yakata Y, Yoshizaki A, Nagayasu T, Sekine I (2005). Expression of p-STAT3 in human colorectal adenocarcinoma and adenoma; correlation with clinicopathological factors. J Clin Pathol.

[29] Bollrath J, Phesse TJ, von Burstin VA, Putoczki T, Bennecke M, Bateman T, Nebelsiek T, Lundgren-May T, Canli O, Schwitalla S, Matthews V, Schmid RM, Kirchner T (2009). gp130-mediated Stat3 activation in enterocytes regulates cell survival and cell-cycle progression during colitis-associated tumorigenesis. Cancer Cell.

[30] Maynard MA, Evans AJ, Hosomi T, Hara S, Jewett MA, Ohh M (2005). Human HIF-3alpha4 is a dominant-negative regulator of HIF-1 and is down-regulated in renal cell carcinoma. FASEB J.

[31] Gyorffy B, Surowiak P, Budczies J, Lanczky A (2013). Online survival analysis software to assess the prognostic value of biomarkers using transcriptomic data in non-small-cell lung cancer. PLoS One.

