# Supplementary material for: HIF-3α1 promotes colorectal tumor cell growth by activation of JAK-STAT3 signaling
Source: Oncotarget. 2016 Feb 9;7(10):11567–79. doi: 10.18632/oncotarget.7272 (PMC4905494; doi:10.18632/oncotarget.7272)
Supplement: Supplementary file 1 [file oncotarget-07-11567-s001.pdf]

HIF-3α1 promotes colorectal tumor cell growth by activation of JAK-STAT3 signaling

Supplementary Materials

Supplementary Table S1: Primers list

QPCR Primer	Sequence (5′–3′)
β-ACTIN F	TATTGGCAACGAGCGGTTCC
β-ACTIN R	GGCATAGAGGTCTTTACGGATGT
HIF-3α F	CCCAGTCGGAGAGTATCGTC
HIF-3α R	GAATGGGTCTGCGAGAGTGT
SOCS3 F	GAGCCAGCGTGGATCTG
SOCS3 R	GGCTCAGCCCCAAGGAC
STAT3 F	CTGCTCCAGGTACCGTGTGT
STAT3 R	CCTCTGCCGGAGAAACAG
IL6 F	GTCAGGGGTGGTTATTGCAT
IL6 R	AGTGAGGAACAAGCCAGAGC
IL6R F	ACTGGTCAGCACGCCTCT
IL6R R	GGGACCATGGAGTGGTAGC
GP130 F	CGGACAGCTTGAACAGAATGT
GP130 R	ACCATCCCACTCACACCTCA
JAK1 F	GAATGACGCCACACTGACTG
JAK1 R	GATGACAAGATGTCCCTCCG
JAK2 F	CCATTCCCATGCAGAGTCTT
JAK2 R	CAGGCAACAGGAACAAGATG

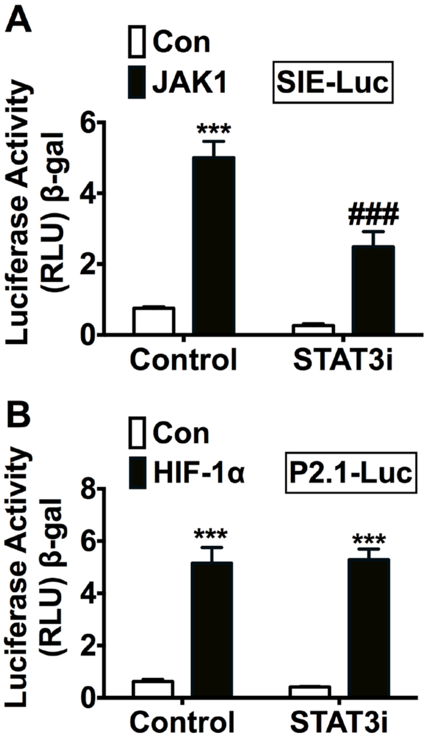

Supplementary Figure S1: STAT3i specifically inhibits the activation of STAT3. (**A**) STAT3 activity luciferase assay in HCT116 CRC cells transfected with JAK1 or control (Con) plasmids and treated with or without STAT3i (100 μM) for 24 hours. (**B**) Enolase promoter (P2.1) luciferase assay in HCT116 CRC cells transfected with HIF-1α or control plasmids and treated with or without STAT3i (100 μM) for 24 hours. ***$p < 0.001$ compared with con plasmid. $^{###}p < 0.001$ compared with untreated control cells.
